# Prognostic factors in adult brainstem glioma: a tertiary care center analysis and review of the literature

**DOI:** 10.1007/s00415-021-10725-0

**Published:** 2021-08-03

**Authors:** Annette Leibetseder, Johannes Leitner, Maximilian J. Mair, Stephan Meckel, Johannes A. Hainfellner, Martin Aichholzer, Georg Widhalm, Karin Dieckmann, Serge Weis, Julia Furtner, Tim von Oertzen, Matthias Preusser, Josef Pichler, Anna Sophie Berghoff

**Affiliations:** 1 Department of Neurology 1, Neuromed Campus, Kepler University Hospital, Johannes Kepler University Linz, Linz, Austria; 2 Department of Biomedical Imaging and Image-Guided Therapy, Medical University of Vienna, Vienna, Austria; 3 Division of Oncology, Department of Medicine I, Medical University of Vienna, Vienna, Austria; 4 Institute of Neuroradiology, Neuromed Campus, Kepler University Hospital, Linz, Austria; 5 Division of Neuropathology and Neurochemistry, Department of Neurology, Medical University of Vienna, Vienna, Austria; 6 Department of Neurosurgery, Neuromed Campus, Kepler University Hospital, Johannes Kepler University Linz, Linz, Austria; 7 Department of Neurosurgery, Medical University of Vienna, Vienna, Austria; 8 Department of Radiooncology, Medical University of Vienna, Vienna, Austria; 9 Division of Neuropathology, Department of Pathology and Molecular Pathology, Neuromed Campus, Kepler University Hospital, Johannes Kepler University Linz, Linz, Austria; 10 Department of Internal Medicine and Neurooncology, Neuromed Campus, Kepler University Hospital, Linz, Austria; 11 Division of Oncology, Department of Medicine I, Medical University of Vienna, Waehringer Guertel 18-20, 1090 Vienna, Austria

**Keywords:** Brainstem glioma, Prognosis, Adult, Primary CNS tumour

## Abstract

**Introduction:**

Adult brainstem gliomas (BSGs) are rare central nervous system tumours characterized by a highly heterogeneous clinical course. Median survival times range from 11 to 84 months. Beyond surgery, no treatment standard has been established. We investigated clinical and radiological data to assess prognostic features providing support for treatment decisions.

**Methods:**

34 BSG patients treated between 2000 and 2019 and aged ≥ 18 years at the time of diagnosis were retrospectively identified from the databases of the two largest Austrian Neuro-Oncology centres. Clinical data including baseline characteristics, clinical disease course, applied therapies, the outcome as well as neuroradiological and neuropathological findings were gathered and analysed. The tumour apparent diffusion coefficient (ADC), volumetry of contrast-enhancing and non-contrast-enhancing lesions were determined on magnetic resonance imaging scans performed at diagnosis.

**Results:**

The median age at diagnosis was 38.5 years (range 18–71 years). Tumour progression occurred in 26/34 (76.5%) patients after a median follow up time of 19 months (range 0.9–236.2). Median overall survival (OS) and progression-free survival (PFS) was 24.1 months (range 0.9–236.2; 95% CI 18.1–30.1) and 14.5 months (range 0.7–178.5; 95% CI 5.1–23.9), respectively. Low-performance status, high body mass index (BMI) at diagnosis and WHO grading were associated with shorter PFS and OS at univariate analysis (*p* < 0.05, log rank test, respectively). ADC values below the median were significantly associated with shorter OS (14.9 vs 44.2 months, *p* = 0.018).

**Conclusion:**

ECOG, BMI, WHO grade and ADC values were associated with the survival prognosis of BSG patients and should be included in the prognostic assessment.

## Introduction

Brainstem gliomas (BSGs) are rare primary tumours of the central nervous system (CNS) in adults accounting only for 1–2% of all primary malignant CNS tumours. In contrast, BSGs are a more common tumour entity in paediatric patients accounting for 10–20% of all brain tumours in children with a peak age of 7–9 years [1–3].

BSGs are characterized by a heterogeneous prognosis with survival times ranging from 1 month to 7 years, resulting in median overall survival (mOS) time of 30–40 months and 5-years survival rates of 45–58% [4–7]. Unlike other tumour entities in children which tend to have better clinical outcomes than adults, those suffering from BSG clinically present homogeneously with quite short mOS rates. The mOS in paediatric BSG ranges from 10 to 12 months with a 5-years survival rate < 5% [8]. Therefore, prognostic assessment is crucial to provide the basis for treatment decisions in this incurable disease. Importantly, treatment modalities have to be chosen with caution due to their localisation to avoid collateral damage resulting from local therapies like radiation or surgery worsening the clinical condition of the patient. Previously, BSGs have been classified according to various aspects including clinicopathologic characteristics, radiographic appearance, tumour location, histologic grading and molecular profiling. In the following, we analysed a cohort from two large tertiary care centres and reviewed the current literature on prognostic parameters in BSG.

### Pathological prognostic factors in brainstem glioma

BSGs categorization is performed by neuropathological grading (II–IV) according to the World Health Organization (WHO). Thereof, WHO grade II gliomas amount to the majority of BSG in adults (70%) compared to their paediatric counterpart, in which reversely WHO Grade IV glioma are more common (50–60%) [9]. Tumour grading (WHO II–IV) significantly impacts clinical outcomes in adult BSGs as overall survival (OS) rates decline with increasing tumour grade. The latter was specified as an independent prognostic factor in several multivariate analysis [5, 6, 10–12].

Established molecular biomarkers in supratentorial glioma, such as IDH-1 mutation and the loss of heterozygosity of 1p19q (LOH 1p19q) are of high importance in the diagnostic and prognostic assessment [4]. Of note, in contrast to WHO II and III supratentorial glial tumours, IDH-1 mutations are suggested to be less common in infratentorial gliomas, even in lower grades (WHO II and III 8%) and LOH 1p19q is almost absent [4, 13]. H3K27m mutation in histone 3 is an important diagnostic and prognostic molecular marker introduced by the WHO classification of 2016 [14]. Its presence defines a distinct tumour entity called “diffuse midline glioma”. Gliomas harbouring this mutation are graded automatically as WHO grade IV tumours and are associated with a poor prognosis. Among adult BSGs, H3K27M mutation was reported as an independent negative prognostic factor (hazard ratio = 3, 95% confidence interval 1.57–5.74) [15].

To further characterize BSGs based on molecular alterations a recent series investigated different methylation patterns in paediatric and adult BSGs [16]. In line with previous attempts in neuro-oncology utilizing methylation characteristics to refine the diagnostic and prognostic discrimination, four distinct subgroups based on the methylation profile could be defined. These clusters termed H3-Pons, H3-Medulla, IDH and PA-like displayed differences in clinical outcome and genomic profiles. Methylation patterns of H3-Pons and H3-Medulla correlated with tumour localization within the pons or medulla, respectively.

### Clinical prognostic parameters in brainstem glioma

Higher age ($\ge$ 40 years) is associated with worse clinical outcome [5, 6, 10], probably attributed to a higher prevalence of high-grade lesions (WHO grade III and IV) in this age group compared to younger patients [6, 17].

A lower Karnofsky Performance Scale (KPS ≤ 70), a duration of symptoms < 3 months and non-Caucasian ethnicity were stated as an unfavourable prognostic factor in several trials as well [5, 6, 10, 17]. A recently published study illustrated an association of direct involvement of cranial nerve V with poor prognosis [18].

### Radiological prognostic factors

Considering radiological factors, BSGs are defined as lesions primarily originating in the brainstem, whereof these tumours are most frequently located in the pons (60%), followed by medulla oblongata (25%) and midbrain (12–15%) [4, 6]. The presence of contrast enhancement or necrosis on magnetic resonance imaging (MRI) were associated with significantly decreased survival rates [6, 10, 19].

Apparent diffusion coefficient (ADC) values are derived from diffusion-weighted tensor imaging (DWI). Several studies reported an inverse correlation between ADC values and tumour cellularity in gliomas, serving as an indicator of tumour cell density and proliferative potential [20, 21]. Thus, quantitative ADC measurements may be used to discriminate between high and low-grade lesions and to predict patients’ outcome [22, 23]. Series of glioma and meningioma reported an association of higher ADC values with better outcome data and vice versa [24–26].

#### Treatment of brainstem glioma

Treatment of BSGs is challenging due to the so far limited evidence mainly based on retrospective case series. Prospective clinical trials dedicated only to BSGs do not exist. In clinical practice, treatment decisions are made in multidisciplinary tumour boards on an individual basis as no international treatment guidelines for BSGs exist.

Maximal safe tumour resection is the first treatment approach as the extent of tumour resection is one of the most important favourable prognostic factor in supratentorial gliomas, however, this approach is not feasible in BSGs [27]. Almost no significant reduction of a tumour mass can be achieved in the brainstem due to its composition of eloquent areas. At best, a stereotactic biopsy of brainstem tumours is feasible by which a definite neuropathological diagnosis can be attained in over 95%. The procedure is stated to be safe with low complication rates resulting in persistent disability and mortality at 1.7% and 0.9% respectively [28]. However, the material available from stereotactic biopsies is diagnostically challenging as heterogeneity of the tumour is not displayed and due to the small amount of material additional molecular work up is complicated or restricted.

Radiation therapy is suggested as first-line therapy in adult BSGs, but ambiguity exists regarding the appropriate irradiation dosage and timing of therapy initiation [10, 17, 29, 30]. According to existing guidelines, radiotherapy should start within 3–5 weeks after surgery/diagnosis with a commonly administered dosage of 50–60 Gy in 1.8–2 Gy daily fractions [31]. Treatment-related toxicities include radiation necrosis, hydrocephalus due to aqueduct stenosis, neurovascular compromise, neuroendocrine deficiency and permanent or transient neurological deficits [32]. In BSGs the role of hypofractionated or proton therapy with the potential advantage to spare structures at higher risk of toxicity from radiotherapy is unknown [31].

Treatment options beyond radiotherapy including chemotherapy are not established. Recently, a study by Panagiotis et al. addressed the role of chemotherapy in combination with radiotherapy in high-grade adult BSGs (WHO III, IV). The results indicated that the addition of chemotherapy to irradiation has a significant positive impact on outcome among WHO grade IV tumours, but not clearly among WHO III tumours [33]. Another retrospective single-arm institutional series of adult BSGs demonstrated a survival benefit for patients with WHO grade III and IV tumours treated in accordance with combined radiochemotherapy followed by adjuvant treatment with temozolomide compared to patients receiving radiation therapy alone [4]. Of note, in H3-K27M-mutant diffuse midline glioma WHO grade IV, the MGMT (O6-methylguanine–DNA methyltransferase) promotor as an established prognostic and predictive biomarker is usually unmethylated indicating less efficacy of alkylating chemotherapy.

The efficacy of antiangiogenic therapies such as bevacizumab is unclear due to the paucity of evidence but might be a therapy strategy for progressive BSGs showing malignant features on MRI. A reduction of tumour volume and improved or maintained KPS after bevacizumab administration was reported in case series and case reports. Importantly, these patients received other therapies additionally, which lowers the probability of a single effect of this agent [34–36].

## Materials and methods

### Patients and data collection

In this study, 34 adult patients (≥ 18 years) with BSGs treated at two neurooncological centres in Austria between 2000 and 2019 were included. For inclusion in this study, primary tumour location in the brainstem (midbrain, pons and/or medulla oblongata) at initial diagnosis was mandatory. Other infratentorial located tumours, which affected dominantly the spinal cord or the cerebellum, or supratentorial tumours with secondary infiltration of the brainstem were excluded. Tumours with components at multifocal regions of the brain, which one of whom sited in the brainstem was one more exclusion criterion. Ependymoma, medulloblastoma and pilocytic astrocytoma were excluded due to their distinct tumour biology.

Clinical data comprised the baseline characteristics of the patients (sex, age at diagnosis, BMI), lesion location, symptoms at initial diagnosis, the clinical course of the disease, neuroimaging, neuropathologic diagnosis, applied treatments including dosage and timing in the first-line setting and at recurrence and clinical outcome. Highly symptomatic disease was defined by the presence of at least two symptoms.

MRI sequences were acquired at two different sites with a 1.5 or 3 Tesla MRI scanner. MR imaging protocols included non-contrast-enhanced and contrast-enhanced T1-weighted images, T2-weighted images and fluid-attenuated inversion recovery (FLAIR) sequences in all cases. Diffusion-weighted images were available in 29 patients at initial diagnosis. The non-contrast-enhanced and contrast-enhanced tumour volume was manually assessed on Flair MR sequences and T1-weighted MR contrast-enhanced sequences, respectively, by two radiologists using the open-source segmentation software ITK-SNAP (version 3.6.0). To obtain the ADC values, tumour regions of interest (ROI) were manually drawn, at each imaging slice, and the minimum (lowest) ADC value of each patient was used for further analysis.

Progression-free survival (PFS) was defined as the period from the date of diagnosis, determined by date of the first MRI showing a brainstem tumour, until radiological progression defined by RANO-criteria and respectively overall survival (OS) until death or last follow up.

Patient data were collected in a password-secured database (FileMaker Pro® Advanced 17, FileMaker Inc., Santa Clara, CA, USA) and were handled anonymously. The local ethic committees of the participating institutions enrolling patients (Vienna, Linz) approved the study (protocol numbers 1166/2019 and 1274/2019).

### Statistical analysis

Descriptive analysis was performed to illustrate the patients clinical and tumour related characteristics. The Kaplan–Meier method was conducted to estimate OS and PFS.

Survival curves among analysed subgroups were compared with the log-rank test. Univariate analyses of different variables were received with 95% confidence intervals (CIs). A *p* value ≤ 0.05 was considered significant. All statistical analyses were computed using IBM SPSS (Version 15).

## Results

### Patients’ characteristics

The median age at initial diagnosis was 38.5 years (range 18–71 years). 16/34 patients (47.1%) were female, 18/34 were male (52.9%), resulting in a female-to-male ratio of 1:1.125. The median BMI of the patients was 25.6 kg/m^2^ (range 17.4–38.1). More details of patients’ characteristics are summarized in Table 1.

**Table 1:** Baseline and treatment characteristics

	*n* = 34	%
Gender		
Male	18	52.9
Female	16	47.1
Median age at diagnosis, years (range)	38.5 (18–71)	
Median BMI at diagnosis, kg/m^2^ (range)	25.6 (17.4–38.1)	
Clinical presentation at diagnosis		
< 2 symptoms	4	11.8
2 or more symptoms	30	88.2
Histological confirmation (biopsy performed)		
Yes	27	79.4
No	7	20.6
Histopathological diagnosis		
WHO II	4	11.8
WHO III	14	41.2
WHO IV	9	26.5
Integrated Diagnosis (WHO 2016)		
Diffuse astrocytoma		
IDH wildtype	2	
IDH mutated	2	
Anaplastic astrocytoma		
IDH wildtype	8	
IDH mutated	2	
Not otherwise specified	4	
Glioblastoma multiforme		
IDH wildtype	1	
IDH mutated	1	
Not otherwise specified	4	
Diffuse midline glioma (H3K17M-mut)	3	
First-line treatment		
Combined radio/chemotherapy	20	58.8
Chemotherapy alone	3	8.8
Radiotherapy alone	5	14.7
Wait and see	2	5.9
No therapy due to rapid disease progression	3	8.8

### Clinical presentation

**Fig. 1:**
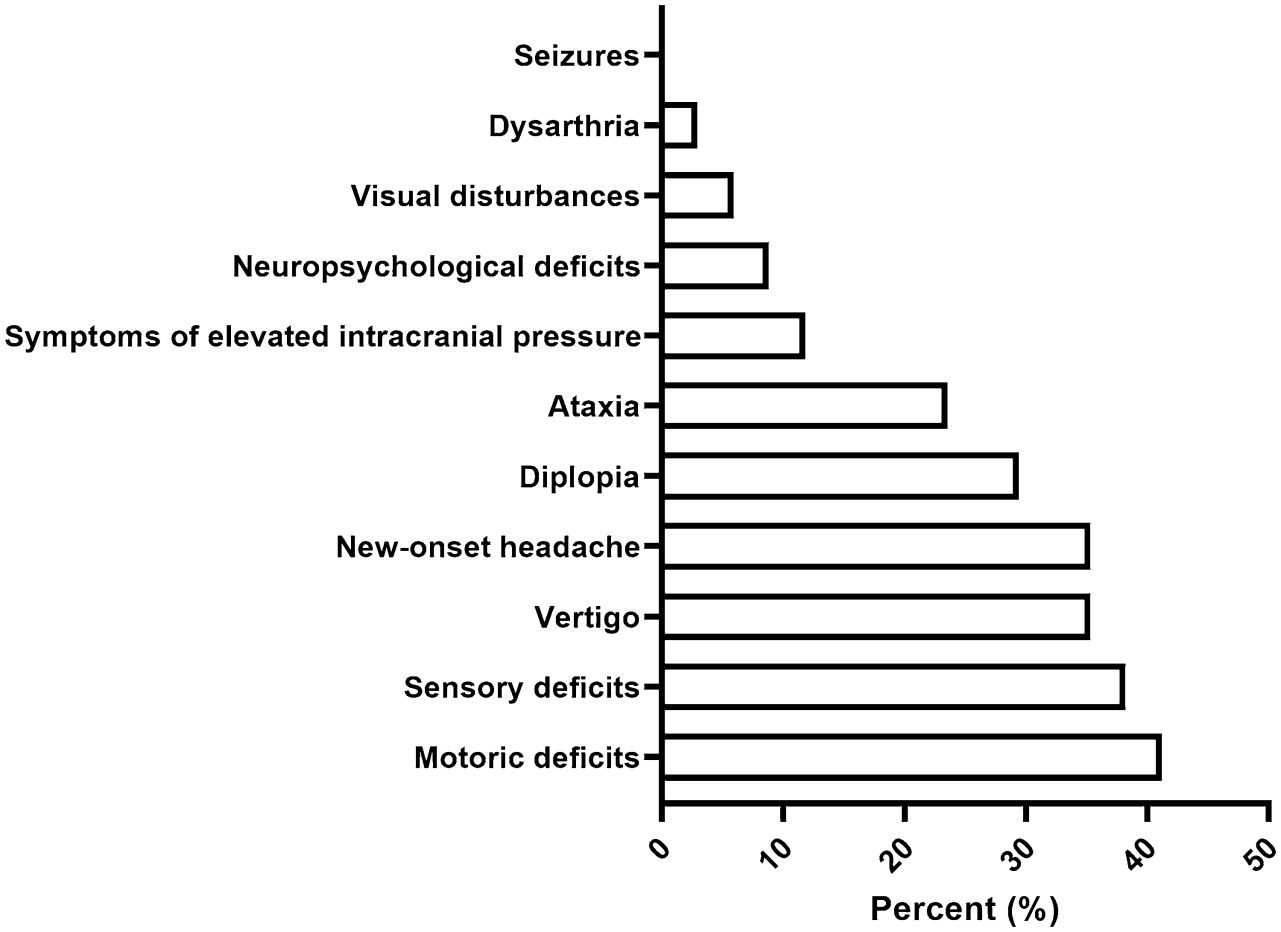
Variety and frequencies (%) of symptoms in the present adult brainstem glioma cohort at diagnosis prior to surgery

Median KPS at diagnosis was 80% (range 50–100%), median ECOG was 1 (range 0–2). Clinical symptoms at presentation pre-surgical included motor deficits (14/34, 41.2%), sensory symptoms (13/24, 38.2%) and headache (12/34, 35.3%), followed by diplopia (10/34, 29.4%), ataxia 8/34 (23.5%), symptoms of elevated intracranial pressure (4/34, 11.8%), neuropsychological deficits (3/34, 8.8%), visual disturbances (2/34, 5.9%) and dysarthria (1/34, 2.9%) (Fig. 1).

The majority of patients showed a combination of symptoms. The median number of symptoms at initial presentation was 2 (range 0–4). 30 patients (88.2%) presented simultaneously with two or more symptoms at first diagnosis. Notably, in one patient (2.9%) diagnosis of brainstem glioma was an incidental finding.

### Pathology characteristics

4 (11.8%) patients were classified to have a WHO grade II, 14 (41.2%) a WHO III and 9 (26.5%) a WHO IV tumour. Molecular workup was not possible in all cases due to low or missing tumour tissue. H3K27M-mutation was available in 8/27 (29.6%) patients. IDH status was obtained in 19/27 (70.4%) cases by immunohistochemistry and sequencing. Results of histopathological diagnosis according to the WHO classification 2016, 4th revised edition [14] of 27/34 (79.4%) patients is outlined in Table 1 and yielded the following results: 2 (5.9%) diffuse astrocytoma IDH-wt, 2 (5.9%) diffuse astrocytoma IDH-mut, 4 (11.8%) anaplastic astrocytoma NOS, 2 (5.9%) anaplastic astrocytoma IDH-mut, 8 (23.5%) anaplastic astrocytoma IDH-wt, 6 (17.6%) GBMs IDH-wt and 3 (8.8%) diffuse midline gliomas (H3 K27M-mut). In seven patients no histological examination was possible. Thus, in these cases diagnosis was based on radiological features only. Regarding the patients without histological workup, positron emission tomography (PET) imaging with 18F-fluoroethyltuorosine (FET) was additionally available in four of seven patients at initial diagnosis. 3 tumours showed increased FET-uptake (SUVmax ranging from 2.1 to 2.9). One tumour which was located in the medulla oblangata showed no metabolic activity.

### Tumour location and radiological characteristics

**Fig. 2:**
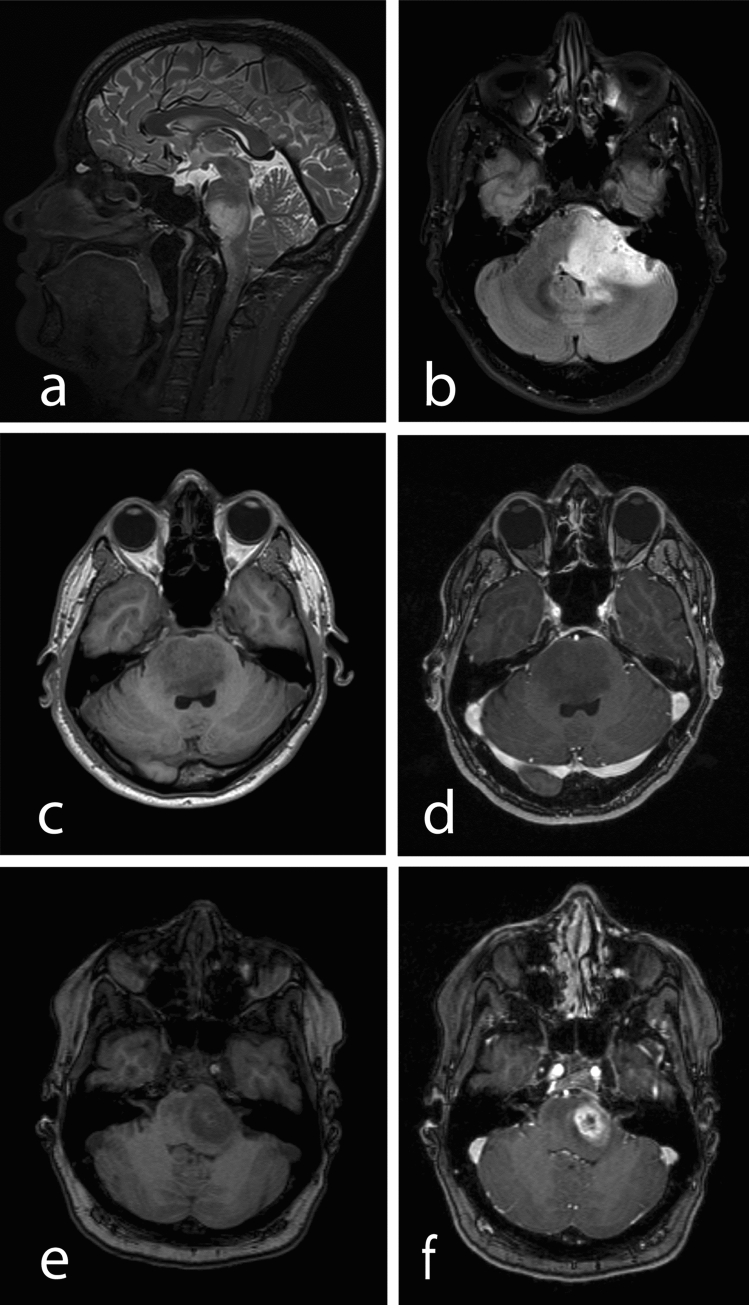
Sagittal T2-weighted MR image representing a brain stem glioma located in the pons and medulla oblongata (**a**) Axial FLAIR image depicting a brain steam glioma located in the pons, the left middle cerebellar peduncle and the left cerebellar hemisphere (**b**) brain stem glioma located in the pons without contrast enhancement on T1-weighted MR images before (**c**) and after (**d**) contrast media application. Brain stem glioma located in the pons with markedly contrast enhancement and central necrosis on T1-weighted MR images before (**e**) and after (**f**) contrast media application

Radiological data were available of 31 patients at initial diagnosis and in addition over the course of the disease as part of follow-up examinations in 15 patients. The tumour location was restricted to the brainstem in 14/31 (45.2%) patients (Fig. 2a), whereas extension into the cerebellum and/or thalamus was observed in 17/31 (54.8%) cases (Fig. 2b).

The tumour was located solely in one part of the brainstem in 6/31 (19.4%) patients, whereof 2 were situated in the mesencephalon, 3 in the pons and 1 in the medulla oblangata. Involvement of two parts of the brainstem occurred in 17/31 (54.8%) patients. Among them, concurrent tumour infiltration of mesencephalon/pons was observed in 11 cases and of medulla oblongata/pons in 6 cases. In the remainder (8/31 patients, 25.8%) tumour was radiologically present in all 3 anatomical parts of the brainstem.

Tumour location and tumour extension into the cerebellum and/or thalamus (tumour location only brainstem vs. involvement of cerebellum and/or thalamus) did not correlate with the symptomatic burden (*p* = 0.242, *χ*^2^-test).

Tumour lesions were contrast-enhanced in 13/31 (41.9%) patients and non-contrast-enhanced in 18/31 (58.1%) patients (Fig. 2c–f). The non-contrast and contrast-enhancing tumour volume varied within a range of 1.947–62.390 cm^3^ and 0.083–13.840 cm^3^ respectively. Radiologically, the majority of the patients (21/31, 67.7%) showed no necrotic parts within the tumour.

### Surgery and first-line treatment

Stereotactic biopsy was performed in 20/34 patients (58.8%), extended biopsy in 4/34 (11.8%) and subtotal resection in 3/34 (8.8%) patients. No patient underwent gross total resection. 7/34 (20.6%) patients had no surgery.

Initial treatments after surgery were chemotherapy with temozolomide, radiation therapy, a combination of both or a wait and see strategy. The majority of patients 20/34 (58.8%) received combined radiochemotherapy, 3/34 (8.8%) patients were treated with chemotherapy alone, 5/34 (14.7%) patients with radiotherapy alone, whereof two of them underwent proton radiotherapy. A wait and see approach was chosen in 2/34 (5.9%) patients. In 3/34 (8.8%) patients adjuvant treatment was planned but was not initiated due to rapid clinical deterioration and disease progression. 1/34 (2.9%) patient was lost to follow-up after surgery.

In total, radiotherapy was completed in 25 patients. The median dosage of radiotherapy was 56 Gy (range 40.05–66 Gy).

### Outcome and survival analysis

Median follow-up time of the whole population was 19 months (range 0.9–236.2 months).

Local tumour progression was seen in 26/34 (76.5%) patients. Among them, eight patients experienced two or more tumour progressions. Median progression-free survival was 14.5 months (range 0.7–178.5 months).

**Fig. 3:**
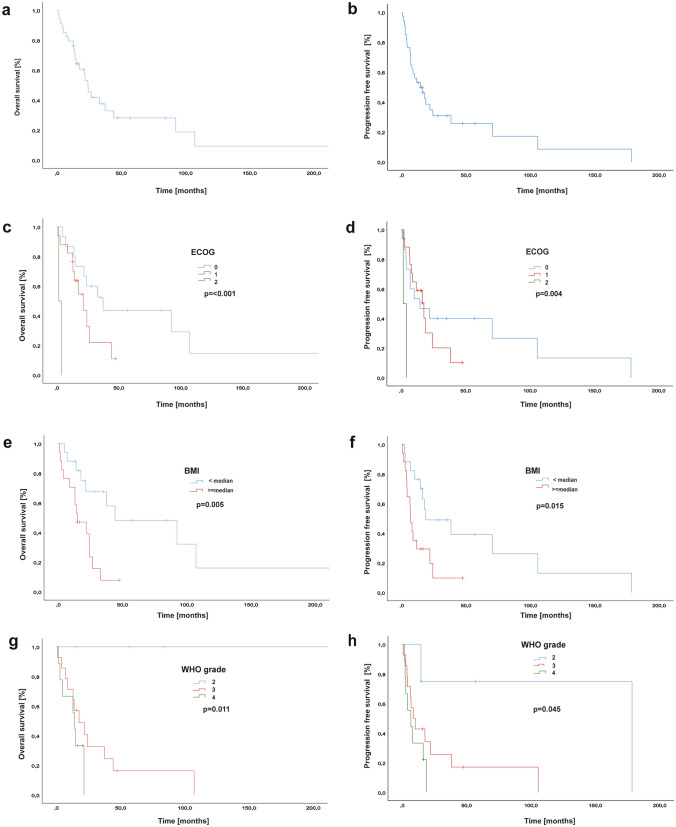
Overall and progression-free survival analysis. **a** OS and **b** PFS of the entire cohort. **c**, **d** OS/PFS according to ECOG at first diagnosis. **e**, **f** OS/PFS according to BMI at initial diagnosis (< median versus ≥ median). **g**, **h** OS/PFS according to WHO grading (II versus III versus IV)

Median overall survival was 24.1 months with 95% CI from 18.078 to 30.12 (1-year-OS-rate 79.4%, 2-year-OS rate 41.2%, 5-years-OS rate 11.8%) (Fig. 3a, b). Patients with lower ECOG (0 vs 1 vs 2) showed significantly longer overall survival rates (median OS: 33.0 vs 21.4 vs 1.6, *p* = 0.000; log-rank test) and progression-free survival rates (median PFS: 10.1 vs 16.3 vs 1.6, *p* = 0.004; log-rank test) than patients with higher ECOG (Fig. 3c, d).

Although not statistically significant, patients with more than two symptoms at initial diagnosis showed a trend towards shorter OS (median OS: 33 vs. 14.1 months, *p* = 0.081; log-rank test) and PFS (16.3 vs. 8.8, *p* = 0.238; log-rank test) than individuals with 0 or only 1 symptom.

Moreover, an age younger than 60 years (age groups 18–39 vs. 40–60 vs. > 60 years) showed a trend towards longer PFS (median PFS: 16.3 vs. 21.9 vs. 4 months, *p* = 0.100; log-rank test) in comparison with elderly patients with a shorter PFS.

Patients with a BMI higher than the median (≥ 25.56 kg/m^2^) had significantly shorter OS (44.2 vs. 14.9 months, *p* = 0.005; log-rank test) and PFS-rates (18.7 vs 7.0 months, *p* = 0.015; log rank-test) (Fig. 3e, f).

The median OS by WHO grade was not reached because all patients with WHO II tumours got censored (*p* = 0.011; log-rank test). The median PFS by WHO grade was as follows: WHO II 178.5 months, WHO III 8.8 months and WHO IV 6.7 months (*p* = 0.045; log rank-test) (Fig. 3g, h).

**Fig. 4:**
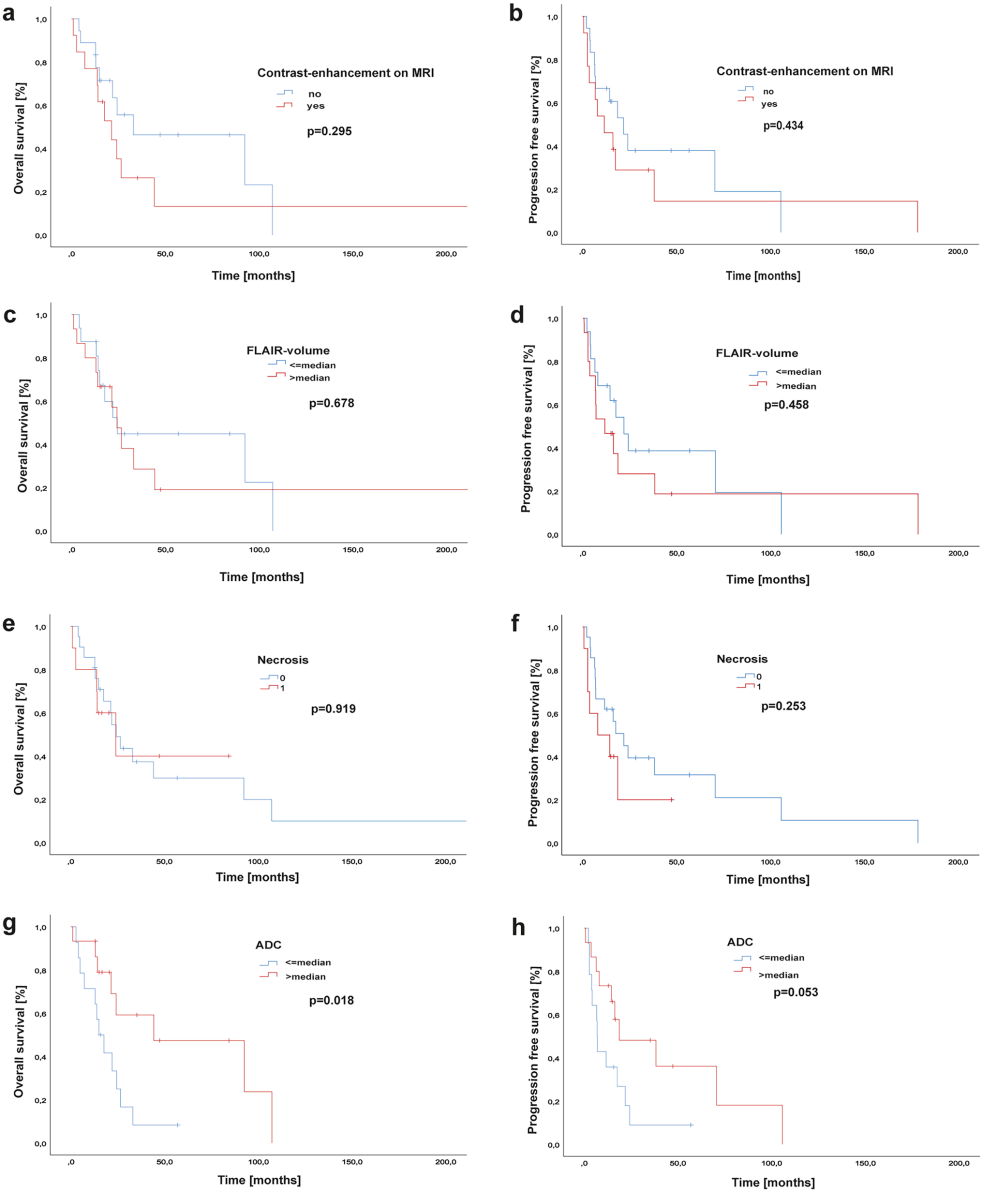
Overall survival and progression-free survival of brainstem gliomas according to radiological characteristics. **a**, **b** Contrast-enhancement (yes versus no). **c**, **d** FLAIR-volume (≤ median vs > median). **e**, **f** Appearance of necrosis on MRI (yes versus no). *g,h* ADC values (≤ median vs > median)

Radiological data including tumour location, the presence of contrast-enhancement or necrosis on MRI and the tumour volume on FLAIR sequences showed no significant results in terms of OS and PFS (*p* > 0.05) (Fig. 4a–f). Nevertheless, higher ADC values than the median (776 10^–6^ mm^2^/s) were significantly associated with longer OS (14.9 vs 44.2 months, *p* = 0.018; log-rank test) and showed a trend towards a longer PFS (6.9 vs 18.7, *p* = 0.053) (Fig. 4g, h). ADC levels did not vary between WHO grading (*p*-value 0.865, *χ*^2^-test).

## Discussion

BSGs in adults represent a rare group of CNS-tumours with varying prognosis and a lack of standardized treatment. Further limitations of surgery due to the localization of these tumours lead to difficulties in reaching a histological and integrated diagnosis according to WHO 2016 classification. In the present study, we sought to investigate clinical and neuroradiological characteristics in a well-defined bicentric BSG cohort aiming to provide prognostic features as support in treatment-decision making. Overall, we observed that WHO grade, ECOG, BMI and ADC-values were significant predictors of survival.

**Fig. 5:**
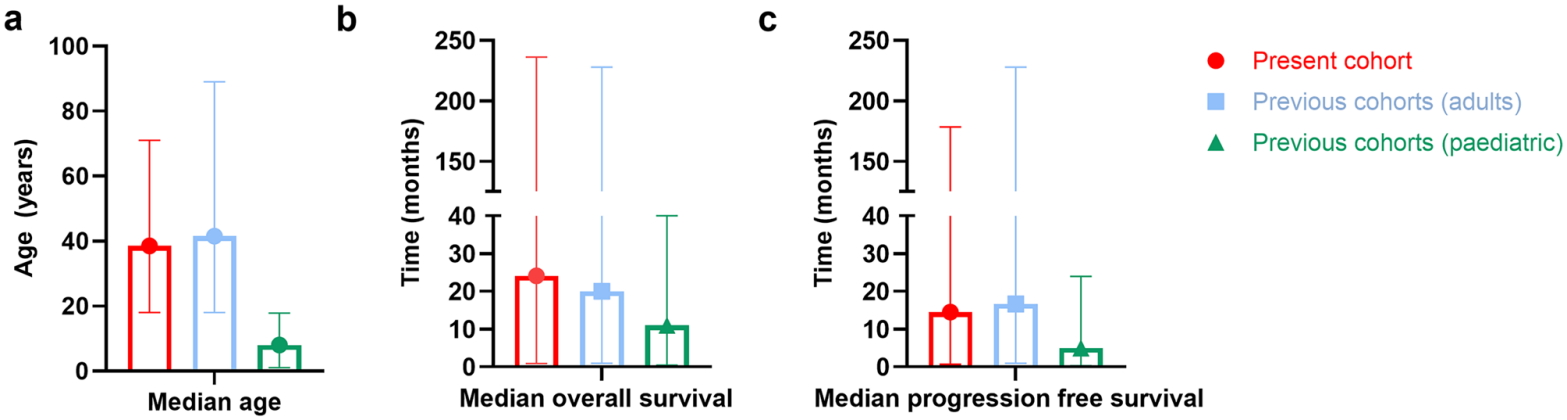
Median age, median overall and progression-free survival and its ranges in the present cohort, previous cohorts (adults) and in children

The median OS of 24.1 months and 5-years-OS rate of 11.8% are more favourable than the one reported in paediatric BSG patients (mOS 10–12 months, 5-years-OS rate < 5%, Fig. 5) [8]. In the present cohort, progression and overall survival times presented with a wide range extending from a few months to years. These results reflect the heterogeneous clinical course of the disease and are in line with existing outcome data of previous studies in adult BSGs (Fig. 5) [4–7]. Previous retrospective series included between 7 and 240 adult BSG patients and reported mOS times from 1 to 85 months (Table 2). Notably, the longest survival data were reported among the studies with an additional enrolment of WHO I tumours [5] and the shortest with the inclusion of only WHO III and IV tumours [12, 37]. Likewise, increasing tumour grade was significantly associated with decreased OS and PFS in our cohort.

**Table 2:** Previous retrospective studies and its results concerning the outcome and prognostic factors

Study	Median age [range]	Time frame	Tumour histology	WHO grade	Molecular markers	Therapy	mPFS [range] (months)	mOS [range] (months)	Prognostic factors (multivariate analysis)
Theeler et al. [4], *n* = 143	36	1990–2012	GBM (28), AA (43), DA (15), gliomas NOS (11), radiographically diagnosed (46)	II–IV	Mutation profiling (9): BRAFV600E (1), 2 PIK3CA mutation (2); immunohistochemistry (IDH1 mutation: 2 of 25 grade II and III tumours, 1 of 17 glioblastomas), MGMT and histone H3.3 not tested	Radiation (118), chemotherapy (27; temozolomide, cytotoxic chemotherapy, and/or bevacizumab)	NA	32.1	Increasing tumour grade (-) Contrast enhancement (-)
Reithmeier et al. [10] *n* = 104	40 [18–89]	1997–2007	DOA (1), AOA (1), DA (23), AA (39), GBM (14), PA (17), EP (2), FA (4);	I (16) II (31) III (42) IV (14)	NA	Radiation (44), radio/-chemotherapy (22), chemotherapy (4), interstitial radiosurgery with brachytherapy (I-125 seeds) (7), or no tumour-specific therapy (23)	NA	18.8	KPS ≤ 70 (–) Age ≥ 40 y (–) Higher tumour grade (III-IV) (–) Radiation therapy or radiochemotherapy ( +)
Dey et al. [12] *n* = 240	48.7	1973–2008	AA (75), GBM (165)	III–IV	NA	Radiation (204; 83,8%), data regarding chemotherapy and other treatment regimens NA	NA	7	Age > 50 years (–) HR 1.98, 95% confidence interval [CI] 1.45–2.70, *p* < 0.001) WHO grade IV (-) HR 1.61, 95% CI 1.15–2.26, p = 0.006 Radiation – no benefit
Hundsberger et al. [7], *n* = 21	41 [20–81]	2004–2012	PA (2), DA (6), AA (7), GBM (6)	I–II (8) III–IV (13)	MGMT promotor methylation (2): negative; IDH1/2 mutation(4): wildtype; P53 mutation (3): 1 wildtype, 2 mutant; 1p19q codeletion (1): intact)	Radiotherapy alone (LG 3, HG 3), radiochemotherapy (LG 2, HG 6), chemotherapy alone (LG 0, HG 2), no postoperative therapy (LG 3, HG 1)	LG 24.1 HG 5.8	LG 30.5 HG 11.5	LG ( +), HG (–)
Babu et al. [37], *n* = 34	42.5 [18–71]	1998–2011	AA (22), GBM (12)	III–IV	IDH mutation (5): 2 mut MGMT promotor methylation (17): 11 positive 1p19q NA; GFAP (28): 28; mean Ki-67 proliferation index: 14%; EGFR expression (15): 14; EGFRvIII expression (16): 2; VEGF (4): 3; Platelet derived growth factor receptor-A (PDGFR-A) (3): 3; PDGFR-B (3): 3; hypoxia-inducible factor 2-alpha (HIF-2-alpha) (1): 1; CAIX (7): 3; KDR (4): 2; HAM56 (7): 4	Radiotherapy (97%), concurrent temozolomide (97%), upon tumour progression: Irinotecan 37%, Lomustinge 14.8%, Etoposide 14.8%, Bevacizumab 33.3%	6.7 III 6.1 IV 6.7	25.8 III 77 IV 12.1	WHO grade IV (-) (HR 4.80; 95% CI 1.86–12.4; *p* = 0.0012 KPS < 80 (–) Duration of symptoms > 2 months ( +)
Babu et al. 53	65 [60–69]	1998–2011	AA (3), GBM (4)	III–IV	Mean Ki-67 proliferation ndex: 9.6 MGMT promotor methylation (5): 5; EGFR (4): 2; EGFRVIII (4): 1	No therapy (1), Radiotherapy and concurrent TMZ (6) Upon tumour progression: Lomustine (3), Bevacizumab (2), Irinotecan (2), Topotecan (1)	6.7 [1.3–13]	13.5 [1.9–45.7]	NA
Reyes-Botero et al. [54], *n* = 17	41 (18–65)	2000–2012	DOA (4), AOA (2), DO (2), AA (2), GBM (3), GBM-PNET (3)	II (6) III (4) IV (7)	Genomic array performed (5 grade III, 6 grade IV); IDH1sequencing (17): 1 IDH1 (R132H) mutation; Histone gene H3F3A encoding H3.3. and HIST13B encoding H3.1. (8): 3 EGFR amplification: 0 BRAF600E mutation: 0	No therapy (2), radiotherapy (9): 5 grade II, 2 grade III, 2 grade IV, concurrent radio-chemotherapy (5): 2 grade III and 3 grade IV), neoadjuvant chemotherapy followed by radiotherapy (1): grade II	31.8 LG 38.1 HG 7.6	48.7 LG 57 HG 16	Higher histological grade (-) Contrast enhancement (-)
Dellaretti et al. [55], *n* = 100 (63 adults, 37 children)	41 (18–75) 6.9 (2–12)	1984–2007	NA	II (49) III + IV (51)		NA	NA	NA	Higher histological grade (-)
Salmaggi et al. [30], *n* = 32	31 (14–78)	1991–2003	PA (2), DA (9), AA (8), GBM (1), Glioma Nos (1)	I-IV	NA	First line strategy: Wait and see (8) Initial treatment (24): Radiotherapy alone (4), concurrent radio-chemotherapy + adjuvant chemotherapy (20: 18 TMZ, 2 PCV) Upon tumour progression: second-line chemotherapy – cisplatin + TMZ (3), PCV (2), TMZ (1), ACNU + procarbazine (1)	10	59	Onset of symptoms/signs and diagnosis ≤ 4 months (-)
Kesari et al. [5], *n* = 101	36 (18–79)	1987–2005	NA, Radiographically diagnosed (47)	I (16) II (15) III (12) IV (3)	NA	First line therapy: Radiotherapy (82), chemotherapy (0) At recurrence: Chemotherapy (40) – PCV, Lomustine, Vincristine, Carboplatin, BCNU, TMZ, Irinotecan; Etoposide, Paclitaxel, Tamoxifen, Hydroxyurea, radiolabelled Anti-EGF antibodies	48 [1–261] I 44 II 48 III 10 IV 10	85 [1–228] I 83 II 168 III 17 IV 16	Non-caucasian ethnicity (-); higher histological grade (-); pontine tumour location (-) vs tumour located at the cervicomedullary junction; age > 40 years (-)
Guillamo et al. [6], *n* = 48	34 (16–70)	1985–1999	PA (1), DA (6), AA (7), GBM (4), DOA (4), DO (1), AO (3), glioma NOS (6)	I (1) II (14) III + IV (17)	NA	First-Line: No therapy (2), radiotherapy (45) At recurrence: chemotherapy – BCNU (12), BCNU + Procarbazine (1), platin-based chemotherapy (12), Ifosfamide (1), ProcarbazineVP16 (1), temozolomide (1)	NA	5.4	Duration of symptoms < 3 months (-) Higher histological grade (-) Necrosis on MRI (-)

Consistent with findings in other glioma subtypes and in previous BSG series [10, 37, 38], lower performance status at initial diagnosis was associated with poor prognosis. However, age as an established prognostic factor in gliomas was not associated with OS and PFS in the present cohort. Therefore, clinical characteristics need to be included in the prognostic assessment of BSG.

Our analysis suggested that BMI at diagnosis correlates with outcome in BSGs. Prior studies regarding BMI as a prognostic factor in glioma are sparse and showed controversial results [39–41], whereby a higher BMI was addressed as an independent prognostic factor in a spectrum of other cancer types including breast cancer, prostate cancer and oral cancer [42–46]. Recent studies postulated an association between pre-diagnostic obesity and poor patient outcome in high-grade gliomas and pilocytic astrocytoma [39, 47]. Several theories of the biology determining the poor prognosis of cancer patients with high BMI were postulated including increased serum insulin-like growth factor-1 and involvement of fatty acid synthase (FASN) pathways [48]. However, so far no biological data exist for glioma.

Prior studies suggested contrast-enhancement and necrosis on imaging as negative prognostic factors in BSGs [4, 6, 9]. By contrast, we did not observe significant differences in outcome regarding these two factors at initial diagnosis in our cohort. A reason for the non-significant findings of contrast-enhancement and necrosis as prognostic factors might be insufficient statistical power due to the small sample size in the present study. Further, an impact on prognosis must be assumed due to the highly diverse molecular landscape of these tumours.

However and consistent with prior findings, lower ADC values below the median were significantly associated with shorter overall survival rates in our analysis. Previous studies in children and in adults reported the correlation between ADC values and outcome [20, 21]. ADC values inversely represent tumour cellularity as the underlying biological condition [24–26]. As cellular density and pleomorphism increases with tumour malignancy, ADC maps were suggested as a useful and non-invasive tool to differentiate between high-grade and low-grade gliomas [49, 50].

There are several limitations when interpreting the available data of our study. The retrospective design has to be taken into account as well as a small sample size, wherefore analysis did not allow to select factors into a multivariable model. Another limitation is the usage of different MRI scans, which potentially limits the accuracy of ADC value analysis [51]. However, a previous study of our group facing a similar limitation could show despite imaging at different scanners an independent prognostic impact of ADC values in single brain metastasis [52].

Molecular data such as IDH mutation and MGMT promotor status as known prognostic and predictive parameters in supratentorial gliomas were not available in all cases. Due to the midline location especially the prevalence of H3K27M-mutation would be of interest as well but was not routinely performed before the year 2016. Further, our cohort also included patients in which biopsy was not feasible and the diagnosis was solely based on radiological features. Hence, in these cases, histological grading and molecular data are totally missing. This scenario occasionally represents a real-life situation and physicians are thrown back to gather clinical and radiological aspects to estimate prognosis and make treatment decisions. However, in histologically verified BSGs adjuvant treatment guidelines do not exist either. This underlines the importance to assess clinical and radiological prognostic features in this tumour entity. Prior retrospective series derived from larger national databases mainly concentrate on survival analysis. In contrast, our study provided real-life data with a high data density of an adult BSG cohort. MRI data at initial diagnosis and a completive follow-up for the whole cohort was available.

In conclusion, the present analysis of a multicentric adult BSG cohort poses the variable clinical course and the challenge of prognostic evaluation in the disease. Our findings underline previously reported prognostic features such as performance status and WHO grading. Further ADC values and BMI were associated with survival prognosis and might be included in the prognostic assessment.

## Data Availability

The datasets generated during and/or analysed during the current study are available from the corresponding author on reasonable request.
